# Simultaneous Assessment of Left Ventricular Volumes and Aortic Valve Annular Dimensions by Three-Dimensional Speckle-Tracking Echocardiography in Healthy Adults from the MAGYAR-Healthy Study—Is There a Relationship?

**DOI:** 10.3390/life15050742

**Published:** 2025-05-06

**Authors:** Attila Nemes, Barbara Bordács, Nóra Ambrus, Csaba Lengyel

**Affiliations:** Department of Medicine, Albert Szent-Györgyi Medical School, University of Szeged, Semmelweis Street 8, P.O. Box 427, H-6725 Szeged, Hungary; bordacs.barbara.aniko@med.u-szeged.hu (B.B.); ambrusnora@gmail.com (N.A.); lecs@in1st.szote.u-szeged.hu (C.L.)

**Keywords:** left ventricular, volume, aortic valve annulus, three-dimensional, echocardiography, speckle-tracking, healthy

## Abstract

**Introduction:** Three-dimensional speckle-tracking echocardiography (3DSTE) can be used to accurately measure the dimensions of the left ventricle (LV) and aortic valve anulus (AVA) at the same time. The present study aimed to conduct an extensive 3DSTE-based investigation of simultaneously assessed end-diastolic and end-systolic LV volumes and AVA dimensions in healthy adults with LVs and AVAs of different sizes. **Methods:** One hundred and seven healthy adults (mean age 35.4 ± 12.2 years, 67 males) were voluntarily enrolled in the present study. **Results:** With increasing end-diastolic AVA area, tendentious increase in both end-diastolic and end-systolic LV volumes could be detected, resulting in preserved LV-EF. With increasing end-systolic AVA area, similar findings were present. Comparing the smaller than mean and the larger than mean end-systolic AVA area subgroups, the end-systolic LV volume proved to be significantly increased in the latter group. With the increase in end-diastolic LV volume, the AVA dimensions remained preserved. With the increase in end-systolic LV volume, only the highest end-systolic LV volume was associated with larger end-systolic AVA area and perimeter; the other parameters remained preserved. In certain circumstances, end-systolic AVA area and perimeter proved to be significantly increased compared to their end-diastolic counterpart. **Conclusions:** With the increase in end-diastolic and end-systolic AVA areas, a tendentious increase in both LV volumes could be detected in healthy adults. Larger end-diastolic LV volume was not associated with dilated AVA dimensions, while larger end-systolic LV volume was associated with dilated end-systolic AVA area and perimeter.

## 1. Introduction

The three-dimensional (3D) and/or speckle-tracking (STE) echocardiographic procedures, which are now considered the most up-to-date in the daily routine, are suitable for the simultaneous non-invasive evaluation of the heart chambers and the valves, allowing even physiologic examinations to be performed [1,2,3]. The combined 3DSTE can be used to accurately measure the dimensions of the left ventricle (LV) and the aortic valve anulus (AVA) at the same time [4,5,6,7,8]. If this possibility is already given, then the question may rightly arise as to what associations can be confirmed between the dimensions of the LV and AVA in the presence of a heart of a different size, taking into account the cardiac cycle. In other words, it can be assessed whether a greater LV is associated with a more dilated AVA, and whether this can be observed in both the diastole and systole. Therefore, the aim of the present study was to conduct an extensive 3DSTE-based investigation by simultaneously assessing end-diastolic and end-systolic LV volumes and AVA dimensions in healthy adults with different LV and AVA sizes.

## 2. Methods

### 2.1. Population of Healthy Adults

One hundred and seven healthy adults (mean age 35.4 ± 12.2 years, 67 males) were voluntarily enrolled in the present study between 2011 and 2017. The subjects were considered to be healthy in the absence of a known disease/disorder or any pathology or other condition, which could affect the findings. None of them were obese, pregnant, smokers, or athletes, and none had practiced yoga 2 weeks before the enrollment. Physical examination, laboratory tests, electrocardiography (ECG), and two-dimensional (2D) Doppler echocardiographic examinations were negative with values in the normal reference ranges in all subjects. In all individuals, routine 2D Doppler echocardiography and 3DSTE-derived data acquisition were established. The acquired 3D echocardiographic datasets were analyzed at a later date. This retrospective study is a part of the ‘Motion Analysis of the heart and Great vessels bY three-dimensionAl speckle-tRacking echocardiography in Healthy subjects (MAGYAR-Healthy) Study, which aimed to compare 3DSTE-derived parameters with other variables in healthy adults among others (’Magyar’ means ’Hungarian’ in the Hungarian language). The study was conducted in accordance with the Helsinki Declaration (revised in 2013); the Institutional and Regional Biomedical Research Committee of the University of Szeged approved the study with a registration number of 71/2011 (the original approval date was issued on 23 May 2011, the latest approval was issued on 17 March 2025). All the participants gave informed consent.

### 2.2. Two-Dimensional Doppler Echocardiography

The Toshiba Artida^TM^ echocardiographic tool (Toshiba Medical Systems, Tokyo, Japan) attached to a PST-30BT (1–5 MHz) phased-array transducer was used for 2D Doppler echocardiography in all cases. The subjects were in left lateral decubitus position, and the transducer was placed on the chest; then, from typical parasternal and apical positions, measurements were performed, including left atrial (LA) and LV chamber quantifications and measurement of LV ejection fraction (EF) using Simpson’s method. Doppler echocardiography was used to exclude significant valvular stenoses and regurgitations and for assessment of transmitral early (E) and late (A) diastolic inflow velocities [1].

### 2.3. Three-Dimensional Speckle-Tracking Echocardiography

First, 3DSTE-derived data acquisitions were established by the same Toshiba cardiac ultrasound machine; then, the transducer was changed to a 3D-capable PST-25SX matrix phased-array transducer (Toshiba Medical Systems, Tokyo, Japan). Following optimization of gain, magnitude, etc., from the apical window, 6 subvolumes within 6 heart cycles during a single breath hold were acquired; the subvolumes were stitched together by the software, automatically creating full-volume, pyramid-shaped 3D echocardiographic datasets. During data analysis, 3D Wall Motion Tracking software (version 2.7, Ultra Extend, Toshiba Medical Systems, Tokyo, Japan) was used [2,4,5,6,7,8].

**Figure 1:**
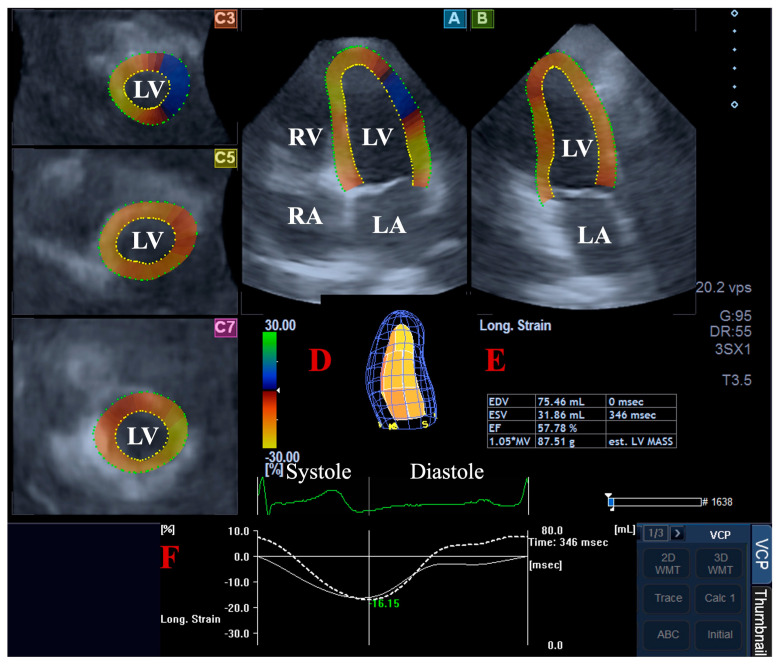
Volumetric assessment of the left ventricle (LV) by three-dimensional (3D) speckle-tracking echocardiography: apical four-chamber (A) and two-chamber long-axis views (B) and short-axis views at basal (C3), midventricular (C5), and apical LV levels (C7) are shown together with a virtual 3D model of the LV (D) and calculated LV volumes (E). Time—global LV circumferential strain curve (white line) and time—LV volume changes curve (dashed white line) are demonstrated as well (F). Abbreviations: LA, left atrium; LV, left ventricle; RA, right atrium; RV, right ventricle; EDV, end-diastolic volume; ESV, end-systolic volume; EF, ejection fraction.

For the LV volumetric assessments, the data were presented in apical long-axis four-chamber (AP4CH) and two-chamber (AP2CH) views and three (basal, midventricular, and apical) cross-sectional views, which were automatically created by the software. Following plane optimalisations and definitions of the mitral annulus—LV septal and lateral edges and the LV apical endocardial surface by the observer—a sequential analysis was performed with automatic contour detection; then, a virtual 3D model of the LV was created together with LV volumetric data with respect to the cardiac cycle [2,4,5,6,7] (Figure 1).

**Figure 2:**
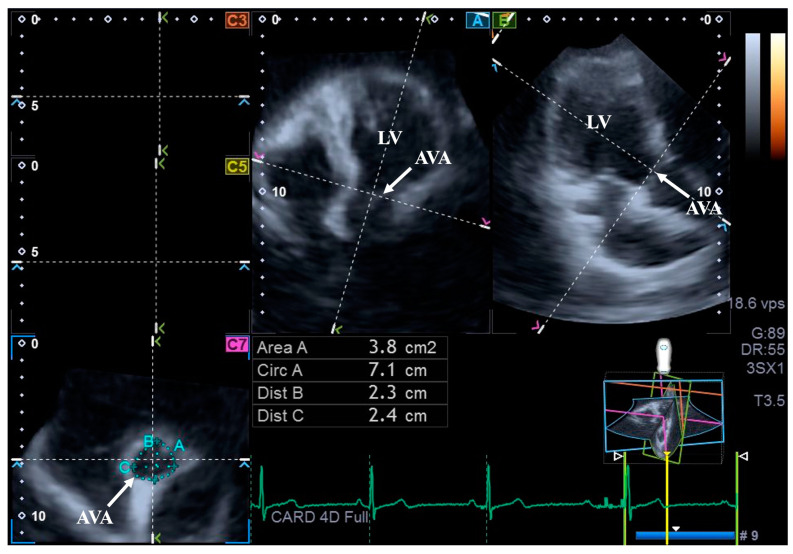
Assessment of the aortic valve annular dimensions by three-dimensional speckle-tracking echocardiography. Abbreviations: LV = left ventricle; AVA = aortic valve annulus; Area = AVA area; Circ = AVA perimeter; Dist B = maximum AVA diameter; Dist C = minimum AVA diameter.

For the assessment of AVA dimensions, longitudinal planes on the AP4CH and AP2CH long-axis views were optimized by tilting these planes so that they were aligned to be parallel to the center line of the aortic root. Then, in the C7 cross-sectional view, the examination plane was aligned perpendicular to the longitudinal plane, to the AVA. These alignments have to be performed carefully to ensure that this C7 plane is perpendicular to the center line and to make measurements on the real AVA, not on the LV outflow tract or on the Valsalva. With this method, the following AVA characteristics were assessed: maximum and minimum AVA diameters (Dmax and Dmin, respectively) and areas (A) and perimeters (P) in the end-diastole (ED) and end-systole (ES) [8] (Figure 2).

### 2.4. Statistical Analysis

Mean ± standard deviation (SD) and n (%) formats were used for continuous or categorical variables, respectively. Statistical significance was considered in the presence of a *p*-value less than 0.05. All analyses were conducted by independent sample *t*-test, analysis of variance (ANOVA), or Kruskal–Wallis H tests, where appropriate. For testing reproducibility of the 3DSTE-derived evaluation of LV volumes and AVA dimensions, the mean ± SD difference in values obtained by two measurements of the same examiner (intraobserver agreement) and by two examiners (interobserver agreement) were tested in 30 healthy subjects together with the respective interclass correlation coefficients (ICCs). SPSS software version 22 (SPSS Inc., Chicago, IL, USA) was used during the statistical analyses.

## 3. Results

### 3.1. Demographic and Clinical Parameters

Height (163.1 ± 9.2 cm), weight (71.4 ± 13.3 kg), heart rate (70.1 ± 1.2 1/s), and systolic and diastolic blood pressures (123.7 ± 3.0 mm Hg and 81.5 ± 1.4 mm Hg, respectively) proved to be in the normal reference ranges in all subjects.

### 3.2. Two-Dimensional Doppler Echocardiographic and 3DSTE Data

The LA diameter measured in the parasternal long-axis view (37.5 ± 3.8 mm), LV end-diastolic diameter and volume (48.3 ± 3.8 mm and 107.2 ± 23.8 mL, respectively), LV end-systolic diameter and volume (32.1 ± 3.3 mm and 38.1 ± 9.2 mL, respectively), thickness of the interventricular septum and LV posterior wall (9.3 ± 1.2 mm and 9.5 ± 1.3 mm, respectively), LV-EF (64.7 ± 3.9%), and transmitral E and A inflow velocities (78.2 ± 16.8 and 59.0 ± 13.6 cm, respectively) proved to be in the normal ranges in all healthy individuals. Valvular regurgitation higher than grade 1 or significant valvular stenosis could not be detected on any of the valves. End-diastolic and end-systolic LV volumes (85.8 ± 21.5 mL and 36.7 ± 10.3 mL, respectively), LV-EF (57.7 ± 5.5%) and LV mass (164.7 ± 31.5 g) as assessed by 3DSTE proved to be normal. 3DSTE-derived end-diastolic and end-systolic AVA-Dmax (2.03 ± 0.31 cm and 2.06 ± 0.29 cm, respectively), AVA-Dmin (1.83 ± 0.29 cm and 1.87 ± 0.28 cm, respectively), AVA-A (3.15 ± 0.85 cm^2^ and 3.37 ± 0.85 cm^2^, respectively) and AVA-P (6.32 ± 0.87 cm and 6.52 ± 0.83 cm, respectively) were in normal ranges, as well.

### 3.3. Classification of Subjects

Healthy individuals were grouped according to their mean ± SD end-diastolic and end-systolic LV volumes and AVA areas based on their lower than mean − SD (64.3 mL, 26.4 mL, 2.28 cm^2^, 2.52 cm^2^, respectively) and higher than mean + SD (107.3 mL, 47 mL, 4 cm^2^, 4.22 cm^2^, respectively) values.

### 3.4. LV Volumes in AVA Dimension Subgroups

With increasing end-diastolic AVA area, a tendentious increase in both end-diastolic and end-systolic LV volumes could be detected, resulting in preserved LV-EF. With increasing end-systolic AVA area, similar findings were present. Comparing the smallest end-systolic AVA area subgroup to the largest end-systolic AVA area subgroup, end-systolic LV volume proved to be significantly increased in the latter group (Table 1).

### 3.5. AVA Dimensions in AVA Dimension Subgroups

With the increase in end-diastolic and end-systolic AVA areas, all end-diastolic and end-systolic AVA maximum and minimum diameters, areas, and perimeters showed significant increase. In certain circumstances, end-systolic AVA area and perimeter proved to be significantly increased compared to their end-diastolic counterpart (Table 1).

### 3.6. AVA Dimensions in LV Volume Subgroups

With the increase in end-diastolic LV volume, the AVA dimensions remained preserved. With the increase in end-systolic LV volume, only the highest end-systolic LV volumes were associated with larger end-systolic AVA area and perimeter; the others remained preserved. In certain circumstances, end-systolic AVA area and perimeter proved to be significantly increased compared to their end-diastolic counterpart (Table 2).

### 3.7. LV Volumes in LV Volume Subgroups

With the increase in end-diastolic LV volume, a significant increase in end-systolic LV volume and LV mass with the preservation of LV-EF could be detected. With the increase in end-systolic LV volume, a significant increase of end-diastolic LV volume and LV mass with reduction of LV-EF were present (Table 2).

### 3.8. Intraobserver and Interobserver Agreements

The values with a mean ± 2 SD difference obtained by two measurements of the same observer and by two observers for the assessments of the 3DSTE-derived LV volumes and AVA dimensions with ICCs are presented in Table 3.

## 4. Discussion

Due to the enormous development of echocardiography, a procedure such as 3DSTE is now available, and it can help in the simultaneous examination of multiple cardiac structures in a simple, easy-to-perform, and easy-to-learn manner [2,4,5,6,7,8]. 3DSTE is validated for LV volumetric measurements, and normal reference ranges for LV volumes are also available [9,10,11,12]. Feasibility and accuracy of 3D echocardiography in the assessment of AVA have also been demonstrated [13]. The significance of simultaneous assessment of certain structures may lie in the fact that even under healthy conditions, the way that the morphological and functional differences of these structures affect each other can be examined. The LV plays a central role in the systemic circulation as a pump, and its ’door’ is the aortic valve [14,15]. A question may therefore rightly arise regarding the potential relationship between the LV’s dimensions characterized by its volumes with respect to the cardiac cycle and the dimensions of the AVA, even in healthy circumstances. According to these facts, 3DSTE-derived LV volumes and AVA dimensions were investigated at the same time using the same 3D echocardiographic datasets, comparing not only the mean, but the smaller than mean and larger than mean values as well.

Several implementations are suggested by the findings of the present study. First of all, 3DSTE was demonstrated to be useful for the quantitative analysis of LV volumes and AVA dimensions with respect to the cardiac cycle at the same time using the same 3D echocardiographic dataset. These findings seem to be very important, because (patho-) physiologic associations between the examined parameters could be examined in an easy-to-perform non-invasive way. Secondly, a number of parameters were used to characterize the AVA, from which the AVA area seemed to be the most important. The AVA is not optimally circle-shaped, but oval in most cases, as shown by diameter data [16]. Thirdly, with the increase in end-diastolic and end-systolic AVA areas, a tendentious (non-significant) increase in both LV volumes could be detected with preserved LV-EF. In other words, as expected, larger valves are associated with larger LV volumes, resulting in normal preserved LV function regardless of the size of the heart. Fourthly, larger end-diastolic LV volume, however, was not associated with dilated AVA dimensions. This result was found in healthy adults. In the presence of various pathologies, LV dilation can be observed, and accordingly, a change in the size of the AVA can be assumed. It would be interesting to examine the above-described relationships in the presence and absence of heart failure in different disorders (e.g., cardiomyopathies, valvular, or congenital heart diseases, etc.). This may be of significance in the grading and possible prognosis of patients, which requires further investigations. Moreover, in a frequent and, in most cases, easy-to-handle disease like hypertension, which is one of the important cardiovascular risk factors, can be considered to be partly responsible for the development of aortic vitium and early atherosclerosis, which changes in LV volumes and the size of the AV and their relationship [17]. Fifthly, larger end-systolic LV volume was associated with dilated end-systolic AVA area and perimeter, suggesting that the AVA may be severely dilated even in healthy circumstances. These findings may raise many new questions about the development of heart failure, which could be a topic of future investigations.

### Limitation Section

The most important limitations are listed here:

- There is a significant difference in quality between images obtained with 2D echocardiography and 3DSTE. Routine 2D echocardiography still provides significantly better image quality and temporal and spatial resolution than that of 3DSTE in a routine ‘average’ subject. The mean frame rate available with 3DSTE is still relatively low (32 ± 2 fps). The size of the 3DSTE-capable transducer is mostly larger and thicker than that used for 2D echocardiography, limiting the optimal positioning of it on the patients’ chest. During digital data acquisitions, for optimal images, one or more (in real-life practice 4–6) subvolume(s) during more than 1 (4–6) cardiac cycle(s) is/are necessary; this increases the probability of the occurrence of potential stitching/motion artifacts during data analysis [2,4,5,6,7]. For optimal images, a stable RR interval on the ECG is also necessary; therefore, in the presence of potential rhythm irregularities or poor image quality, subjects had to be excluded from 3DSTE-derived analysis.
- Not only the LV, but the right ventricle and both atria can also be examined at the same time together with other valvular annuli using the same acquired 3D echocardiographic dataset [18,19,20]. However, the present study did not aim to perform further measurements.
- The use of other echocardiographic techniques to characterize valvular function was not an aim of this study [21].
- All the individuals were healthy, although potential latent diseases could not be ruled out with 100% certainty.
- Some differences between parameters proved to be non-significant or tendentious. These findings must of course be treated in their proper context and interpreted accordingly.

## 5. Conclusions

With the increase in end-diastolic and end-systolic AVA areas, a tendentious increase was detected in the case of both LV volumes in healthy adults. Larger end-diastolic LV volume was not associated with dilated AVA dimensions, while larger end-systolic LV volume was associated with dilated end-systolic AVA area and perimeter.

## Figures and Tables

**Table 1:** Aortic valve annular dimensions and left ventricular volumetric parameters in different aortic valve annular groups.

	ED-AVA-A ≤ 2.3 cm^2^ (n = 18)	2.3 cm^2^ < ED- AVA-A < 4 cm^2^ (n = 70)	ED-AVA-A ≥ 4 cm^2^ (n = 19)	ES-AVA-A ≤ 2.52 cm^2^ (n = 16)	2.52 cm^2^ < ES- AVA-A < 4.22 cm^2^ (n = 73)	ES-AVA-A ≥ 4.22 cm^2^ (n = 18)
**LV volumes**						
**ED-LV volume (mL)**	76.3 ± 13.2	87.0 ± 22.3	90.2 ± 22.4	76.1 ± 19.6	85.4 ± 21.1	91.7 ± 23.1
**ES-LV volume (mL)**	33.5 ± 6.9	37.0 ± 10.5	38.8 ± 11.3	31.6 ± 8.1	36.3 ± 9.8	41.1 ± 12.4 **
**LV-EF (%)**	56.0 ± 5.6	58.2 ± 5.5	57.2 ± 5.3	58.2 ± 5.5	58.1 ± 5.7	55.7 ± 4.9
**LV mass (g)**	157.8± 31.2	165.8 ± 31.2	167.4 ± 32.3	153.4 ± 33.1	165.3 ± 29.7	168.3 ± 35.8
**Aortic valve annular dimensions**						
**ED-AVA-Dmax (cm)**	1.6± 0.2	2.0 ± 0.2 *	2.4 ± 0.2 */†	1.7 ± 0.2	2.0 ± 0.2 **	2.4 ± 0.3 **/††
**ED-AVA-Dmin (cm)**	1.4 ± 0.1	1.8 ± 0.2 *	2.2 ± 0.2 */†	1.5 ± 0.2	1.8 ± 0.2 **	2.2 ± 0.2 **/††
**ED-AVA-A (cm^2^)**	1.9 ±0.3 ‡	3.1 ± 0.4 */‡	4.5 ± 0.5 */†	2.2 ± 0.5	3.1 ± 0.6 **/‡	4.2 ± 0.8 **/††/‡
**ED-AVA-P (cm)**	5.0± 0.5 ‡	6.3 ± 0.4 */‡	7.6 ± 0.4 */†	5.2 ± 0.6	6.3 ± 0.7 **/‡	7.3 ± 0.7 **/††/‡
**ES-AVA-Dmax (cm)**	1.7 ± 0.2	2.1± 0.2 *	2.4 ± 0.3 */†	1.7 ± 0.2	2.0 ± 0.2 **	2.5 ± 0.2 **/††
**ES-AVA-Dmin (cm)**	1.5 ± 0.2	1.9± 0.2 *	2.2± 0.2 */†	1.5 ± 0.2	1.8 ± 0.2 **	2.2 ± 0.2 **/††
**ES-AVA-A (cm^2^)**	2.4 ± 0.4	3.3 ± 0.6 *	4.4 ± 0.8 */†	2.1 ± 0.4	3.3 ± 0.4 **	4.8 ± 0.5 **/††
**ES-AVA-P (cm)**	5.5 ± 0.5	6.5 ± 0.6 *	7.5 ± 0.7 */†	5.2 ± 0.5	6.5 ± 0.5 **	7.8 ± 0.5 **/††

* *p* < 0.05 vs. ED-AVA-A ≤ 2.3 cm^2^; † *p* < 0.05 vs. 2.3 cm^2^ < ED-AVA-A < 4 cm^2^; ** *p* < 0.05 vs. ES-AVA-A ≤ 2.52 cm^2^; †† *p* < 0.05 vs. 2.52 cm^2^ < ES-AVA-A < 4.22 cm^2^; ‡ *p* < 0.05 vs. end-systolic counterpart. Abbreviations. ED = end-diastolic, ES = end-systolic, AVA = aortic valve annulus, Dmax = maximum AVA diameter, Dmin = minimum AVA diameter, A = AVA area, P = AVA perimeter.

**Table 2:** Aortic valve annular dimensions and left ventricular volumetric parameters in different left ventricular volumetric parameter groups.

	ED-LV Volume ≤ 64.3 mL (n = 13)	64.3 mL < ED-LV Volume < 107.3 mL (n = 83)	ED-LV Volume ≥ 107.3 mL (n = 11)	ES-LV Volume ≤ 26.4 mL (n = 15)	26.4 mL < ES-LV Volume < 47 mL (n = 77)	ES-LV Volume ≥ 47 mL (n = 15)
**LV volumes**						
**ED-LV volume (mL)**	52.7 ± 14.0	84.9 ± 11.8 *	125.3 ± 16.1 */†	64.4 ± 12.5	83.5 ± 15.2 **	121.9 ± 18.4 **/††
**ES-LV volume (mL)**	24.5 ± 5.0	35.7 ± 7.1 *	55.2 ± 8.8 */†	22.5 ± 2.7	36.3 ± 5.9 **	55.1 ± 8.0 **/††
**LV-EF (%)**	57.8 ± 6.1	58.0 ± 5.7	56.0 ± 4.1	64.4 ± 5.3	56.7 ± 4.7 **	54.6 ± 4.0 **/††
**LV mass (g)**	131.8 ± 24.5	163.4 ± 27.1 *	203.9 ± 28.3 */†	138.8 ± 28.6	163.3 ± 26.8 **	202.6 ± 26.2 **/††
**Aortic valve annulus**						
**ED-AVA-Dmax (cm)**	2.1 ± 0.3	2.0 ± 0.3	2.1 ± 0.3	2.0 ± 0.2	2.0 ± 0.3	2.1 ± 0.3
**ED-AVA-Dmin (cm)**	1.8 ± 0.3	1.8 ± 0.3 ‡	1.9 ± 0.2	1.8 ± 0.3	1.8 ± 0.3	1.9 ± 0.3
**ED-AVA-A (cm^2^)**	3.1 ± 0.8	3.1 ± 0.9 ‡	3.4 ± 0.7	3.1 ± 0.6	3.2 ± 0.8 ‡	3.5 ± 0.7 ‡
**ED-AVA-P (cm)**	6.3 ± 0.8	6.3 ± 0.9 ‡	6.6 ± 0.6	6.3 ± 0.7	6.3 ± 0.9 ‡	6.7 ± 0.7 ‡
**ES-AVA-Dmax (cm)**	2.0 ± 0.3	2.1 ± 0.3	2.1 ± 0.3	2.0 ± 0.3	2.1 ± 0.3	2.1 ± 0.3
**ES-AVA-Dmin (cm)**	1.8 ± 0.3	1.9 ± 0.3	1.9 ± 0.2	1.8 ± 0.3	1.9 ± 0.3	2.0 ± 0.2
**ES-AVA-A (cm^2^)**	3.2 ± 0.8	3.4 ± 0.9	3.7 ± 0.7	3.2 ± 0.8	3.3 ± 0.9	3.8 ± 0.7 **
**ES-AVA-P (cm)**	6.3 ± 0.8	6.5 ± 0.9	6.8 ± 0.7	6.3 ± 0.8	6.5 ± 0.8	7.0 ± 0.6 **

* *p* < 0.05 vs. ED-LV volume ≤ 64.3 mL, † *p* < 0.05 vs. 64.3 mL < ED-LV volume < 107.3 mL; ** *p* < 0.05 vs. ES-LV volume ≤ 26.4 mL; †† *p* < 0.05 vs. 26.4 mL < ES-LV volume < 47 mL; ‡ *p* < 0.05 vs. end-systolic counterpart. Abbreviations. ES = end-systolic, ED = end-diastolic, AVA = aortic valve annulus, Dmax = maximum AVA diameter, Dmin = minimum AVA diameter, A = AVA area, P = AVA perimeter.

**Table 3:** Intraobserver and interobserver agreement in measurement of three-dimensional speckle-tracking echocardiography-derived left ventricular volumes and aortic valve annular dimensions.

	Intraobserver Agreement		Interobserver Agreement	
	Mean ± 2SD Difference in Values Obtained by 2 Measurements of the Same Observer	ICC Between 2 Measurements of the Same Observer	Mean ± 2SD Difference in Values Obtained by 2 Observers	ICC Between Independent Measurements of 2 Observers
**ED-LV volume (mL)**	1.7 ± 6.3	0.90 (*p* < 0.01)	1.7 ± 4.2	0.90 (*p* < 0.01)
**ES-LV volume (mL)**	0.8 ± 4.9	0.90 (*p* < 0.01)	1.0 ± 4.3	0.90 (*p* < 0.01)
**ED-AVA-Dmax (cm)**	−0.05 ± 0.18	0.86 (*p* < 0.01)	−0.04 ± 0.18	0.88 (*p* < 0.01)
**ED-AVA-Dmin (cm)**	−0.02 ± 0.22	0.90 (*p* < 0.01)	−0.04 ± 0.18	0.93 (*p* < 0.01)
**ED-AVA-A (cm^2^)**	−0.15 ± 0.59	0.93 (*p* < 0.01)	−0.11 ± 0.55	0.95 (*p* < 0.01)
**ED-AVA-P (cm)**	−0.04 ± 0.65	0.92 (*p* < 0.01)	−0.13 ± 0.62	0.92 (*p* < 0.01)
**ES-AVA-Dmax (cm)**	0.01 ± 0.27	0.93 (*p* < 0.01)	0.04 ± 0.30	0.94 (*p* < 0.01)
**ES-AVA-Dmin (cm)**	0.04 ± 0.32	0.83 (*p* < 0.01)	0.05 ± 0.30	0.83 (*p* < 0.01)
**ES-AVA-A (cm^2^)**	0.16 ± 0.71	0.92 (*p* < 0.01)	0.11 ± 0.71	0.93 (*p* < 0.01)
**ES-AVA-P (cm)**	−0.02 ± 0.52	0.91 (*p* < 0.01)	0.02 ± 0.55	0.93 (*p* < 0.01)

Abbreviations. ED = end-diastolic, ES = end-systolic, AVA = aortic valve annulus, Dmax = maximum AVA diameter, Dmin = minimum AVA diameter, A = area, P = perimeter.

## Data Availability

The original contributions presented in the study are included in the article; further inquiries can be directed to the corresponding author.

## References

[1] Lang R.M., Badano L.P., Mor-Avi V., Afilalo J., Armstrong A., Ernande L., Flachskampf F.A., Foster E., Goldstein S.A., Kuznetsova T. (2015). Recommendations for cardiac chamber quantification by echocardiography in adults: An update from the American Society of Echocardiography and the European Association of Cardiovascular Imaging. J. Am. Soc. Echocardiogr..

[2] Franke A., Kuhl H.P. (2003). Second-generation real-time 3D echocardiography: A revolutionary new technology. MedicaMundi.

[3] Levy P.T., Machefsky A., Sanchez A.A., Patel M.D., Rogal S., Fowler S., Yaeger L., Hardi A., Holland M.R., Hamvas A. (2016). Reference Ranges of Left Ventricular Strain Measures by Two-Dimensional Speckle-Tracking Echocardiography in Children: A Systematic Review and Meta-Analysis. J. Am. Soc. Echocardiogr..

[4] Ammar K.A., Paterick T.E., Khandheria B.K., Jan M.F., Kramer C., Umland M.M., Tercius A.J., Baratta L., Tajik A.J. (2012). Myocardial mechanics: Understanding and applying three-dimensional speckle tracking echocardiography in clinical practice. Echocardiography.

[5] Urbano-Moral J.A., Patel A.R., Maron M.S., Arias-Godinez J.A., Pandian N.G. (2012). Three-dimensional speckle-tracking echocardiography: Methodological aspects and clinical potential. Echocardiography.

[6] Muraru D., Niero A., Rodriguez-Zanella H., Cherata D., Badano L. (2018). Three-dimensional speckle-tracking echocardiography: Benefits and limitations of integrating myocardial mechanics with three-dimensional imaging. Cardiovasc. Diagn. Ther..

[7] Gao L., Lin Y., Ji M., Wu W., Li H., Qian M., Zhang L., Xie M., Li Y. (2022). Clinical Utility of Three-Dimensional Speckle-Tracking Echocardiography in Heart Failure. J. Clin. Med..

[8] Nemes A., Ambrus N., Lengyel C. (2025). Does left ventricular rotational mechanics depend on aortic valve annular dimensions in healthy adults?—A three-dimensional speckle-tracking echocardiography-derived analysis from the MAGYAR-Healthy Study. Biomedicines.

[9] Nesser H.J., Mor-Avi V., Gorissen W., Weinert L., Steringer-Mascherbauer R., Niel J., Sugeng L., Lang R.M. (2009). Quantification of left ventricular volumes using three-dimensional echocardiographic speckle tracking: Comparison with MRI. Eur. Heart J..

[10] Kleijn S.A., Brouwer W.P., Aly M.F.A., Russel I.K., de Roest G.J., Beek A.M., van Rossum A.C., Kamp O. (2012). Comparison between three-dimensional speckle-tracking echocardiography and cardiac magnetic resonance imaging for quantification of left ventricular volumes and function. Eur. Heart J. Cardiovasc. Imaging.

[11] Kleijn S.A., Aly M.F.A., Terwee C.B., van Rossum A.C., Kamp O. (2012). Reliability of left ventricular volumes and function measurements using three-dimensional speckle tracking echocardiography. Eur. Heart J. Cardiovasc. Imaging.

[12] Kormanyos A., Kalapos A., Domsik P., Gyenes N., Lengyel C., Nemes A. (2021). Normal reference values of left ventricular volumetric parameters in healthy adults-real-life single-center experience from the three-dimensional speckle-tracking echocardiographic MAGYAR-Healthy Study. Quant. Imaging Med. Surg..

[13] Tamborini G., Fusini L., Muratori M., Cefalù C., Gripari P., Ali S.G., Pontone G., Andreini D., Bartorelli A.L., Alamanni F. (2014). Feasibility and accuracy of three-dimensional transthoracic echocardiography vs. multidetector computed tomography in the evaluation of aortic valve annulus in patient candidates to transcatheter aortic valve implantation. Eur. Heart J. Cardiovasc. Imaging.

[14] Caro C.C., Pedley T.J., Schroter R.C., Seed W.A. (1978). The Mechanics of Circulation.

[15] Berman M.N., Tupper C., Bhardwaj A. (2022). Physiology, Left Ventricular Function. StatPearls.

[16] Anderson R.H. (2000). Clinical anatomy of the aortic root. Heart.

[17] Wu X., Sha J., Yin Q., Gu Y., He X. (2025). Global burden of hypertensive heart disease and attributable risk factors, 1990–2021: Insights from the global burden of disease study 2021. Sci. Rep..

[18] Nemes A., Kormányos Á., Rácz G., Ruzsa Z., Achim A., Ambrus N., Lengyel C. (2023). Tricuspid annular and right atrial volume changes are associated in healthy adults-insights from the three-dimensional speckle-tracking echocardiographic MAGYAR-Healthy Study. Front. Cardiovasc. Med..

[19] Nemes A., Kormányos Á., Ambrus N., Lengyel C. (2022). Associations between Mitral Annular and Left Atrial Volume Changes in Healthy Adults-Detailed Analysis from the Three-Dimensional Speckle-Tracking Echocardiographic MAGYAR-Healthy Study. Rev. Cardiovasc. Med..

[20] Kitano T., Nabeshima Y., Nagata Y., Takeuchi M. (2023). Prognostic value of the right ventricular ejection fraction using three-dimensional echocardiography: Systematic review and meta-analysis. PLoS ONE.

[21] Naguib K.I., Attia M.A., Bashandy M.S., Reihan M.S., Dabash T.A., El-Salam A.B.A., Helal H.H., Bahbah E.I. (2021). The role of trans-thoracic echocardiography in the assessment of aortic annular diameter. Medicine.

